# Epidemiological features of lung giant cell carcinoma and therapy for patients with *EGFR* mutations based on case reports and the surveillance, epidemiology, and end results (SEER) database

**DOI:** 10.18632/oncotarget.15831

**Published:** 2017-03-02

**Authors:** Shan-Shan Weng, Ying Cao, Xiu-Jun Tang, Li-Zhen Zhu, Yi-Nuo Tan, Cai-Xia Dong, Jia-Qi Chen, Hong Shen, Ying Yuan

**Affiliations:** ^1^ Department of Medical Oncology, The Second Affiliated Hospital, Zhejiang University School of Medicine, Hangzhou, Zhejiang Province, China

**Keywords:** lung giant cell carcinoma, EGFR, EGFR-TKIs

## Abstract

Epidermal growth factor receptor-tyrosine kinase inhibitors (*EGFR*-TKIs) are the standard first line treatment for advanced non-small cell lung cancer (NSCLC) with sensitive *EGFR* mutations. Among NSCLC, giant cell carcinoma of the lung (GCCL) is a rare pathological subtype with poor prognosis, with no confirmed evidence about its epidemiological features or therapeutic efficiency of *EGFR*-TKIs. We present two advanced GCCLs with sensitive *EGFR* mutations, also collected the cases of GCCL from our hospital and the Surveillance, Epidemiology, and End Results (SEER) program. Kaplan-Meier methods and Cox proportional hazards modeling were used to perform the survival analyses. Both two cases of advanced GCCL with sensitive *EGFR* mutations benefited from *EGFR*-TKIs. Twelve GCCLs were recorded in our hospital from May 2006 to July 2015. GCCL is associated with males (83.3%) and smoking status (63.6%). The *EGFR* mutation rate was 40.0%. In SEER database, the total number of GCCLs was 184, 0.11% for all NSCLCs. In Kaplan-Meier analysis, the 5-year overall survival of GCCL patients was significantly lower than that of non-GCC NSCLC (16% and 19%; *P*<0.001), and it was confirmed in multivariate analysis. Further survival analyses indicated that male were more susceptible to GCCL and GCCL was prone to metastasize. Only age and M stage were independent prognostic factors for GCCL in the multivariate analysis. In conclusion, GCCL was an unfavorable prognostic factor and associated with males and metastasis. GCCL patients with sensitive *EGFR* mutations may also benefit from *EGFR*-TKI, we therefore recommend the evaluation of EGFR in the treatment of advanced GCCL.

## INTRODUCTION

Lung cancer is still considered a fatal disease worldwide [1]. The American cancer prediction report in 2015 indicated that lung cancer ranked second in terms of the morbidity rate and first in terms of the mortality rate in both gender groups [2]. Once patients are diagnosed with metastatic lung cancer, medical treatment is believed to be the main method for prolonging life, and surgical intervention is no longer an option. According to the results of a myriad of studies, epidermal growth factor receptor (*EGFR*) mutations play an important role in selecting treatment options for advanced non-small cell lung cancer (NSCLC) patients. Epidermal growth factor receptor-tyrosine kinase inhibitors (*EGFR*-TKIs) have become the standard first choice for advanced NSCLC patients with sensitive *EGFR* mutations. Data show that the progression-free survival (PFS) of advanced NSCLC patients with sensitive *EGFR* mutations treated with *EGFR*-TKIs as the first-line treatment could reach 9.5-13.7 months, which is much longer than that achieved by traditional chemotherapy (4.6-6.9 months), and the overall efficiency rate of *EGFR*-TKIs was much higher than for traditional chemotherapy (58%-84% vs 15%-47%) [3–8]. Giant cell carcinoma of the lung is a rare pathological type of NSCLC, and it is subcategorized as pulmonary sarcomatoid carcinoma (PSC). PSC is a group of poorly differentiated sarcoma-containing or sarcomatoid (shuttle shape and/or giant cell)-differentiated non-small cell cancers which comprising the following four subtypes: pleomorphic carcinoma, spindle cell carcinoma, sarcomatoid carcinoma and pulmonary mother cell carcinoma [9]. Compared with other NSCLC types, giant cell carcinoma of the lung has poor prognosis [9, 10]. However, there is no confirmed evidence describing giant cell carcinoma of the lung in terms of its epidemiological features, including the *EGFR* mutation rate, or therapeutic efficiency to *EGFR*-TKIs of patients with sensitive *EGFR* mutations. Our current study aims to discuss these issues based on case reports and the Surveillance, Epidemiology, and End Results (SEER) database. Informed consent was obtained.

## RESULTS

### Two cases

**Figure 1:**
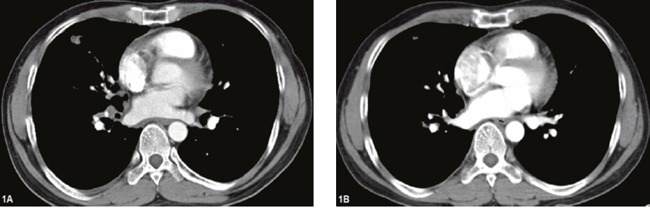
Chest computed tomography of the patient (case 1) before and after gefitinib **(A)** tumor mass in right lung on Mar 24^th^, 2015 (before gefitinib): **(B)** tumor mass in right lung on Apr 22^th^, 2015 (one month after gefitinib).

**Figure 2:**
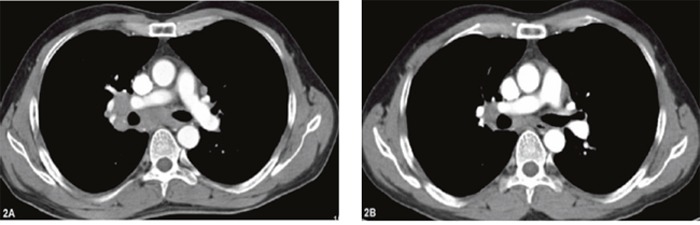
Chest computed tomography of the patient (case 1) before and after gefitinib **(A)** lymph node metastasis in lung hilum and mediastinum on Mar 24^th^, 2015 (before gefitinib); **(B)** lymph nodes shrinking on Apr 22^th^, 2015 (one month after gefitinib).

Case 1 A 46-year-old male was admitted to our hospital (Second Affiliated Hospital of Zhejiang University School of Medicine in Hangzhou) to be treated for persistent respiratory distress in March, 2015. Serum carcinoembryonic antigen (CEA) was elevated to 29.6 ng/ml (normal range: <5 ng/mL), and serum cell keratin 211 increased up to 65.1 ng/mL (normal range: <5 ng/mL). Physical examination revealed an enlarged left supraclavicular lymph node, which was 2.5 cm * 3.0 cm, hard in consistency, immobile and ill defined. Enhanced chest computed tomography (CT) showed a mass in the right middle lobe (approximately 16.7 mm * 13.6 mm), and there were multiple lymph node metastases in bilaterally supraclavicular areas, the mediastinum and the right hilum. There was segmental atelectasis in the right inferior lobe with accompanying pleural effusion (Figure 1A and 2A). No other metastatic evidence was observed through general assessment. The pathology based on ultrasound-guided coarse needle biopsy of the left supraclavicular lymph node showed a metastatic and poorly differentiated tumor, indicating metastatic GCCL. The results of immunohistochemistry were as follows: Napsin A -, CD56 -, Syn -, CgA -, TTF-1 -, CK7 +, Ki-67 50% +, P53 -, P63 -, CK5/6 -, CD68 -, EMA +, CEA +, SMA -, and ALK -. Thus, the diagnosis was GCCL, and there was bilateral supraclavicular lymph node metastasis. Furthermore, the detection of *EGFR* mutations revealed a deletion mutation of exon 19. This patient received oral gefitinib (a type of *EGFR*-TKI) treatment (0.25 g once a day) beginning in Mar 29, 2015. After one month of treatment, the respiratory distress improved, and the lymph node in the left supraclavicular area could no longer be identified. Tumor markers, including CEA and cell keratin 211, gradually decreased to the normal range. Enhanced chest CT was repeated in Apr 22, 2015 (Figure 1B and 2B), which showed the masses shrank compared with the former picture. The therapeutic effect evaluation was partial remission (PR).

The patient continues to receive oral gefitinib treatment (performance status (PS) = 0) and a continued PR therapeutic effect is observed. Only minor adverse drug reactions were reported, including dental ulcers and swelling of the gums, skin rash, etc. This patient was treated at the follow-up clinic regularly and achieved more than 13 months of PFS with TKI treatment as the first-line therapy.

**Figure 3:**
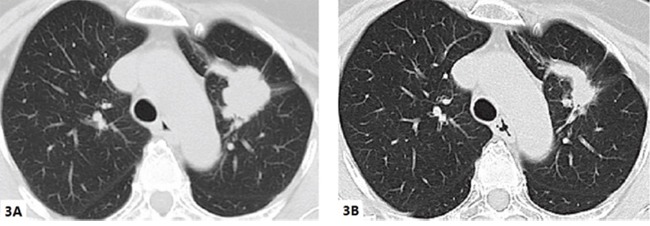
Chest computed tomography of the patient (case 2) before and after icotinib **(A)** tumor mass in left lung on Aug 26^th^, 2014 (before icotinib): **(B)** tumor mass in the left lung on Oct 13^th^, 2014 (one and a half month after icotinib).

**Figure 4:**
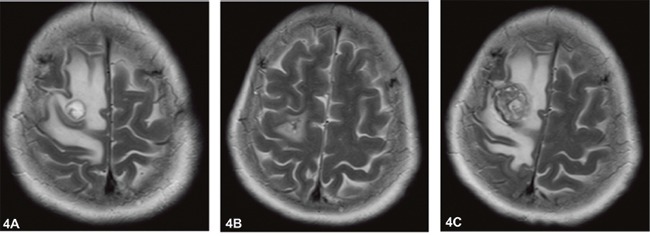
Magnetic resonance imaging of brain showed the mass located in the frontal lobe for Case 2 **(A)** Aug 27, 2014: at initial diagnosis; **(B)** Oct 16, 2014: after the first surgery; and **(C)** Feb 3, 2015 at the time of postoperative recurrence.

Case 2 A 57-year-old female was admitted to our hospital complaining of left limb weakness and reduced mobility for 1 week. Physical examination was only positive with left lower limb power 4/5. Cranial CT scan and enhanced magnetic resonance imaging (MRI) revealed multiple lesions in the brain that were highly indicative of metastases. Chest CT scan identified space-occupying lesions in the upper lobe of the left lung (size approximately 3.6 cm * 2.9 cm * 3.1 cm), which were indicative of lung cancer (Figure 3A). Positron emission tomography/computed tomography (PET/CT) further identified the diagnosis of left lung cancer with right frontal lobe and right iliac metastases. Because the massive frontal lobe lesion caused obvious compression symptoms (Figure 4A), the patient consented to undergo microscopic resection of the lesion under general anesthesia on Aug 29^th^, 2014. The pathology of the sample collected in the operation was reported as metastatic carcinoma, indicating metastatic GCCL. The results of immunohistochemistry were as follows: TTF-1 +, CK(AE1/AE3) partly +, CK7 partly +, CD68 -, GFAP -, Ki67 70-80%, EMA +, SMA tumor cell -, Desmin -, CEA several + and P53 +. The status of *EGFR* mutations was further detected, showing an exon 21 mutation, a missense mutation named L858R. Then, the patient began to receive oral icotinib target treatment (0.125 g, three times a day). The patient refused whole brain radiotherapy. Enhanced chest CT after one and a half months of treatment (Oct 13^th^, 2014) suggested that the lung mass was significantly diminished (Figure 3B). Enhanced cranial MRI (Oct 16th, 2014) showed postoperative changes after the first surgery (Figure 4B). The therapeutic effect evaluation at this point was PR. Fortunately, there were no obvious adverse drug reactions and no drug resistance developed in the course of treatment.

After four months, following icotinib treatment (Feb 3, 2015), the patient again presented with left lower limb weakness. Enhanced cranial MRI showed postoperative recurrence of metastatic carcinoma in the right frontal lobe of the brain (Figure 4C). The PFS of the first-line icotinib treatment was 4.3 months. On the seventh day after admission to our hospital, muscle power on the left side was distinctly decreased. Considering the limitations of conservative treatment as well as respecting the preference of both the patient and her relatives, the patient again underwent resection of the right frontal lobe lesion on Feb 6^th^, 2015. Postoperative pathology revealed metastatic and poorly differentiated carcinoma in the right frontal lobe of the brain, indicating metastatic GCCL. The results of immunohistochemistry were as follows: TTF-1 +, CK7 -, CK (AE1/AE3) +, GFAP -, NSE -, CgA -, Syn -, CD68 +, CD163 +, CD56 -, and Ki-67 30% +. Unfortunately, this patient was lost to follow-up after the second operation.

### GCCL cases recorded in our hospital from may 2006 to July 2015

To investigate the epidemiological features, including the mutation rate of *EGFR*, and the therapeutic efficiency of *EGFR*-TKIs in GCCL patients with sensitive *EGFR* mutations, we collected the cases of GCCLs recorded in our hospital from May 2006 to July 2015 (Table 1). Factors such as the age, sex, smoking status, date of diagnosis, final diagnosis, diagnosed procedure, EGFR mutation and treatment were described. Finally, twelve patients were screened, and we further detected the status of *EGFR* mutations. Demographically, ten out of twelve were males, accounting for 83.3% of the group. Seven patients admitted to smoking, and there was a positive rate of 63.6% (except one was unknown). Two had metastatic GCCLs. Ten out of twelve were diagnosed by surgery, while two were diagnosed by biopsy(including case 1). Regarding the status of the *EGFR* mutations, four out of ten had *EGFR* mutations (though two samples were lost), including two for exon 19 deletion and two for exon 21 mutation. The rate of *EGFR* mutations was 40.0%. Additionally, six patients had wild-type *EGFR* mutations. Of those patients with mutated *EGFR*, only two patients received *EGFR*-TKIs treatment, and these were the two cases we reported above.

**Table 1:** The patients diagnosed GCCL in our hospital from May 2006 to July 2015

No.	Sex	Age	Smoking history	Date of diagnosis	Diagnosis	Diagnostic procedure	*EGFR* mutation	*EGFR*-TKI
1	M	59	>30 years	2006-05-02	GCCL	Surgery	Wild	N
2	M	76	>10 years	2008-11-07	GCCL	Surgery	Wild	N
3	M	76	Denied	2007-02-01	GCCL	Surgery	Exon 19del	N
4	M	74	>30 years	2010-11-29	GCCL	Surgery	Wild	N
5	M	69	Unknown	2012-05-04	GCCL	Surgery	No	N
6	M	32	Denied	2013-08-15	GCCL	Surgery	Wild	N
7	M	79	>30 years	2013-08-29	GCCL		Wild	N
8	F	48	Denied	2014-01-30	GCCL	Surgery	Exon 21L858R	N
9	M	67	>30 years	2014-08-07	GCCL	Biopsy	No	N
10*	M	46	>20 years	2015-03-27	GCCL with metastasis	Biopsy	Exon 19del	Y
11	M	64	>40 years	2015-07-03	GCCL	Surgery	Wild	N
12*	F	57	Denied	2014-09-03	GCCL with metastasis	Surgery	Exon 21L858R	Y

*: patients reported in our cases

Abbreviations: GCCL: giant cell carcinoma of lung; No: without genetic detection; N: without the treatment of *EGFR*-TKIs; Y: received the treatment of *EGFR*-TKIs.

### Data from the surveillance, epidemiology, and end results program

**Figure 5:**
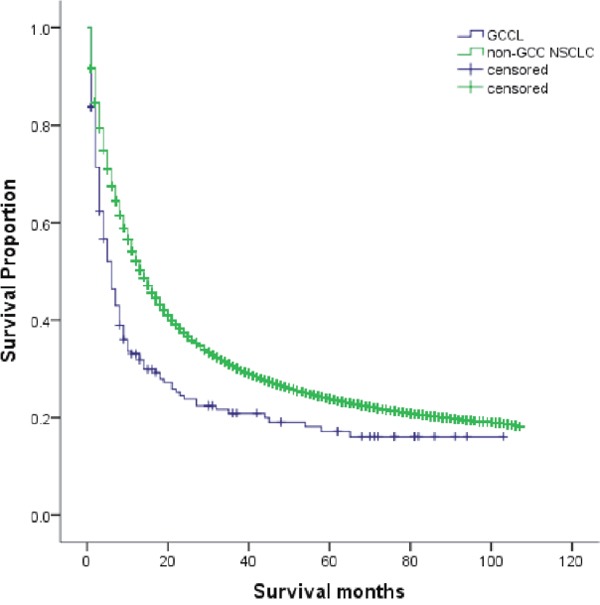
Kaplan-Meier curve of overall survival in patients with GCCL and with non-GCC NSCLCs (*P* <0.001) GCCL: giant cell carcinoma of lung; NSCLC: non-small cell lung cancer

We identified 172, 913 NSCLCs, including 184 GCCLs, during the period of 2004 to 2010 from the SEER database. The incidence of GCCL was 0.11% among all NSCLCs. Kaplan-Meier curve analysis showed that the 5-year overall survival (OS) of GCCL was significantly lower than that of non-GCC NSCLCs (16% and 19%, respectively; *P*<0.001; Figure 5; Table 2), and the median OS (mOS) was 6.0 months (95% confidence interval [CI] 4.399-7.601) compared to 14.0 months (13.860-14.140) with *P* value <0.001. In multivariate analysis, GCCL was an independent unfavorable prognostic factor (hazard ratio [HR] 1.510, 95% CI 1.281-1.780; P<0.001).

**Table 2:** Survival analysis by Kaplan-Meier method in patients with GCCL and non-GCC NSCLC (The *P value was <0.001)

	mOS* (months)	95% CI(months)
GCCL	6.0	4.399-7.601
Non-GCC NSCLC	14.0	13.860-14.140

Abbreviations: mOS: median overall survival; CI: confidence interval.

To investigate the epidemiological features of GCCL, we made further survival analyses among GCCL (Table 3). Male patients appeared to be more susceptible to GCCL (63.0%) and GCCL was prone to metastasize (60.3%). Univariate analysis revealed that the age, N stage, T stage, M stage, American Joint Committee on Cancer (AJCC) 7th stage and methods of diagnostic confirmation were statistically correlated to the GCCL prognosis, with *P* values < 0.05 (Table 4). However, among all of the above variables, only age and M stage were independent prognostic factors for GCCL in the multivariate analysis (HR 1.934, 95% CI 1.195-3.129, *P*=0.007; and HR 4.600, 95% CI 2.937-7.205, *P*<0.001, respectively).

**Table 3:** Demographics and characteristics of giant cell carcinoma of lung in the surveillance, epidemiology, and end results database in our study

Demographic/characteristic	N
Age (years)	
<75	141 (76.6%)
≥75	43 (23.4%)
Sex	
Male	116 (63.0%)
Female	68 (37.0%)
Race	
White	152 (82.6%)
Black	22 (12.0%)
Other	10 (5.4%)
Tumor location	
Main bronchus	8 (4.3%)
Upper lobe	97 (52.7%)
Middle lobe	9 (4.9%)
Lower lobe	40 (21.7%)
Overlapping lesion	2 (1.1%)
T stage	
T1	27 (14.7%)
T2	55 (29.9%)
T3	46 (25.0%)
T4	25 (13.6%)
N stage	
N0	69 (37.5%)
N1	18 (9.8%)
N2	62 (33.7%)
N3	18 (9.8%)
M stage	
M0	73 (39.7%)
M1	111 (60.3%)
AJCC 7^th^ stage	
I	22 (12.0%)
II	27 (14.7%)
III	16 (8.7%)
IV	111 (60.3%)
Diagnostic confirmation	
Only exfoliative cytology	24 (13.0%)
Only radiography	2 (1.1%)
Histology	136 (73.9%)
Unknown	22 (12%)

Abbreviations: AJCC: American Joint Committee on Cancer.

**Table 4:** Univariate analysis of factors influencing mOS by Kaplan-Meier method in GCCL

Variable	mOS (months)	95% CI (months)	*P* value
Age (years)			0.005
<75	7.0	5.240-8.760	
≥75	4.0	2.176-5.824	
Sex			0.281
Male	6.0	4.005-7.995	
Female	6.0	3.882-8.118	
Race			0.949
White	5.0	3.138-6.862	
Black	8.0	5.586-10.414	
Other	6.0	0.000-13.321	
Tumor location			0.294
Main bronchus	NA	NA	
Upper lobe	NA	NA	
Middle lobe	NA	NA	
Lower lobe	NA	NA	
Overlapping lesion	NA	NA	
T stage			0.003
T1	18.0	10.015-25.985	
T2	8.0	4.499-11.501	
T3	5.0	1.845-8.155	
T4	4.0	1.150-6.850	
N stage			0.021
N0	10.0	3.994-16.006	
N1	6.0	4.108-7.892	
N2	5.0	3.132-6.868	
N3	6.0	2.241-9.759	
M stage			<0.001
M0	23.0	7.209-38.791	
M1	3.0	2.040-3.960	
AJCC 7^th^ stage			<0.001
I	65.0	NA	
II	21.0	10.242-31.758	
III	14.0	1.387-26.613	
IV	3.0	2.040-3.960	
Diagnostic confirmation			0.001
Only exfoliative cytology	4.0	0.799-7.201	
Only radiography	1.0	1.000-1.000	
Histology	6.0	3.782-8.218	
Unknown	NA	NA	

Abbreviations: NA: not available; mOS: median overall survival; GCCL: giant cell carcinoma of lung; CI: confidence interval.

## DISCUSSION

Giant cell carcinoma of the lung was first discovered and named by Nash and Stout in 1958 [11]. With the increasing number of cases reported, GCCL was found to be more likely to metastasize and patients deteriorated more rapidly than those with other NSCLC subtypes [12]. GCCL accounts for 0.1-0.4% of the total incidence of lung cancers [13]. Patients with respiratory symptoms are more likely to seek medical attention [14, 15]. GCCL predominantly affects male patients with a long smoking history [14, 16, 17]. From a histopathological point of view, GCCL almost exclusively consists of giant cells, and it is easily distinguished from adenocarcinoma, squamous cell cancer and large cell carcinoma. The definitive diagnosis of GCCL relies on the histopathology evaluation of the resected tumor instead of cytology and small biopsy specimens [9, 18]. Nevertheless, it was not adopted in the latest WHO classification of tumors of the lung published in 2015, as the immunophenotype and genetic background of GCCL were relatively distinct and no absolute obstacle exists in the diagnosis of biopsy samples for experienced pathologists. Both of our patients were diagnosed according to the 2004 World Health Organization classification of lung cancers. In accordance with other NSCLC subtypes, surgical resection is still the primary option for treating GCCL. However, many patients lost the chance to undergo surgery because they already had metastasis at the time of diagnosis, and their median survival time was 8.0-10.0 months [13]. Unfortunately, previous retrospective studies and case reports suggested GCCL is not sensitive to chemotherapy [19]. Vieira et al investigated the efficacy of first-line chemotherapy in 97 patients with advanced lung sarcomatoid carcinomas. The result of their research revealed that, at the first evaluation after chemotherapy, 69% of patients were classified as progressive disease (PD), while 31% of patients achieved disease control though only half achieved PR. In survival analyses, the median PFS (mPFS) was 2.0 months (95% CI: 1.8–2.3) and the mOS was 6.3 months (95% CI: 4.7–7.8) [20]. Our two patients presented with metastases at the time of diagnosis. One experienced only respiratory distress, while the other, by contrast, presented with metastatic symptoms at the initial diagnosis. Because *EGFR* mutations were detected in both patients, *EGFR*-TKIs were employed, instead of conventional chemotherapy, as the initial treatment regimen. Currently, the patient described in case 1 has a PFS of more than 13 months and the patient in case 2 reached a 4.3-month PFS. The outcomes of our patients are more optimistic than those presented in Vieria et al's study. Yosuke et al evaluated the efficacy of molecular targeted therapy for advanced pulmonary pleomorphic carcinoma. One patient harboring EGFR exon 19 deletion was treated with gefitinib, and then achieved a complete response of about 35 months [21]. We have reason to believe that targeted therapy might be effective treatment for GCCL. We therefore recommend the evaluation of EGFR in the treatment of advanced GCCL.

*EGFR*-TKIs have become the standard first-line therapy for advanced lung adenocarcinoma patients with *EGFR* mutations [22]. According to recent studies, *EGFR* mutations have ethnical distinctions. Up to 57.9% of NSCLC patients with *EGFR* mutations were identified in an Asian cohort, [23] and the frequencies of *EGFR* mutations in non-Asian cohorts ranged from 7.0% to 33.2% [24–26]. Erlotinib and gefitinib are the most commonly used *EGFR*-TKIs. Additionally, lcotinib (Conmana) is a type of *EGFR*-TKI that was developed and approved in China, and it is used to treat locally advanced or metastatic NSCLC with EGFR mutations. A randomized, double blinded and phase III study (ICOGEN) compared the efficacy and adverse effects of icotinib and gefitinib in treating advanced NSCLC that is unresponsive to a platinum-based chemotherapy regimen. Icotinib was found to be as effective as gefitinib in terms of the mPFS (4.6 months [95% CI 3.5-6.3] vs 3.4 months [2.3-3.8]; *P*=0.13), objective response rate (ORR) (62.1% vs 53.8%; *P*=0.49), mOS (13.3 months [95% CI 11.1-16.2] vs 13.9 months [11.^4^–^17^.^3^]; *P*=0.57) and adverse effects [27]. NSCLCs with *EGFR* mutations have a 70-80% responsive rate to *EGFR*-TKIs [18, 24, 28, 29]. GCCL was reported to have a lower rate of *EGFR* mutation compared with other NSCLC subtypes [17]. Moreover, the efficacies of *EGFR*-TKI for treating GCCL or pulmonary sarcomatoid carcinoma (PSC) have not yet been disclosed due to their low morbidity rates. In our study, only four patients carried *EGFR* mutations (although two samples were missing), and the *EGFR* mutation rate was 40%. Among the four GCCLs cases with positive *EGFR* mutations, the two patients presented in our report received and benefited from *EGFR*-TKIs. However, the other two patients were either lost to follow-up or developed brain metastasis and declined the *EGFR*-TKI intervention. As a result, a definite conclusion can hardly be draw on the use of *EGFR*-TKIs for treating GCCL with *EGFR* mutations. Zou et al diagnosed a PSC (cT3N2M0, stage IIIa) with a wild-type *EGFR* gene. The patient refused surgery and had reached complete remission of the lung mass after receiving radiotherapy and chemotherapy. However, metastases were later discovered in the para-aortic lymph nodes, bilateral iliac fossa and right gluteal region. An *EGFR* exon 21 L858R gene mutation was identified after biopsy of the right gluteal region metastasis. Then, this patient was treated with erlotinib and had a 6-month PFS before the appearance of metastases. It is noteworthy that the new metastases were subsequently identified to have a wild-type *EGFR* gene [30]. This case demonstrated the *EGFR* mutational heterogeneity in PSC, which may lead to its resistance to *EGFR*-TKIs. Our two patients had GCCL with *EGFR* mutations and responded well to *EGFR*-TKI treatment in the beginning. In our first case, the lesions progressively shrank according to radiological imaging and a valuable indicator, CEA, was continuously declining as well. Therefore, it is sensible to expect that gefitinib will remain effective in the near future. Nevertheless, the patient in the second case had a 4.3-month PFS after icotinib treatment, but the patient experienced treatment failure after presenting with new brain metastases. Then, we detected *EGFR* mutations from her second resected lesion, and the *EGFR* exon 21 L858R gene mutation was still identified. As a result, considering the reasons for *EGFR*-TKI failure, an insufficient concentration of icotinib in the brain and the lack of brain radiotherapy may result in an negative outcome. However, another explanation could be drug resistance. Regrettably, this patient was lost to follow-up after her second surgery. The 4.3-month PFS achieved by icotinib treatment is comparable to that of conventional chemotherapy in GCCL. Additionally, *EGFR*-TKI treatment has fewer adverse effects, and it has been validated in several phase 3 trials [4–7]. According to these clinical trials, compared with patients treated with standard chemotherapy, the skin toxicity (mainly rash, up to 71.1%), abnormal liver transaminases and diarrhea were more frequent, but myeloid suppression (including neutropenia and anemia, alopecia, fatigue, and appetite loss) and other severe adverse events were less common in the *EGFR*-TKIs group. With respect to the adverse reactions of our patients to *EGFR*-TKI treatment, the first patient merely suffered from mild aphthous ulcers and skin rash, and the second patient tolerated the treatment without any significant side effects.

In conclusion, GCCL was found to be an unfavorable prognostic factor that has a tendency to affect males and to metastasize. Similar to other NSCLC subtypes, advanced GCCL with sensitive *EGFR* mutations can be treated with *EGFR*-TKIs as the first-line therapy. Moreover, extensive research on GCCL is required to validate the incidence of *EGFR* mutations and their response rate to *EGFR*-TKI treatment.

## MATERIALS AND METHODS

### Case reports

First, we report two cases of advanced giant cell carcinoma of the lung patients with sensitive *EGFR* mutations. These two patients received *EGFR*-TKI treatment. We performed a follow-up with the patients to discuss the survival benefits. Then, we collected the cases of GCCLs recorded in our hospital from May 2006 to July 2015 and determined their *EGFR* mutation status to study their epidemiological features, including the *EGFR* mutation rate and the therapeutic efficiency of *EGFR*-TKIs for patients with sensitive *EGFR* mutations. Informed consent was obtained.

### Data from the surveillance, epidemiology, and end results program

Considering the small sample at our hospital, we retrieved data from the SEER program to further investigate the characteristics of GCCL. The inclusion criteria are individuals older than 18 years who were diagnosed with NSCLC between January 1, 2004 and December 31, 2010. Patients whose OS rates were less than one month or died of secondary cancer were excluded from our study. Those cases were reclassified according to the criteria of the AJCC 7th edition. Finally, we identified 172, 913 NSCLCs in the period of 2004 to 2010 from the SEER program, including 184 GCCLs. The patients’ demographic and tumor factors, including age, sex, race, tumor location, TNM stage and diagnostic confirmation, were described. Statistical analyses were performed using statistical software package SPSS version 19.0. Kaplan-Meier methods with the log-rank test and Cox proportional hazards modeling were used to perform the survival analyses. *P* <0.05 was considered statistically significant.

## References

[1] Allemani C, Weir HK, Carreira H, Harewood R, Spika D, Wang XS, Bannon F, Ahn JV, Johnson CJ, Bonaventure A, Marcos-Gragera R, Stiller C, Silva GAE (2015). Global surveillance of cancer survival 1995-2009: analysis of individual data for 25 676 887 patients from 279 population-based registries in 67 countries (CONCORD-2). Lancet.

[2] Siegel RL, Miller KD, Jemal A (2015). Cancer statistics, 2015. CA Cancer J Clin.

[3] Sonnenblick A, de Azambuja E, Azim HA, Piccart M (2015). An update on PARP inhibitors--moving to the adjuvant setting. Nat Rev Clin Oncol.

[4] Maemondo M, Inoue A, Kobayashi K, Sugawara S, Oizumi S, Isobe H, Gemma A, Harada M, Yoshizawa H, Kinoshita I, Fujita Y, Okinaga S, Hirano H (2010). Gefitinib or chemotherapy for non-small-cell lung cancer with mutated EGFR. N Engl J Med.

[5] Mitsudomi T, Morita S, Yatabe Y, Negoro S, Okamoto I, Tsurutani J, Seto T, Satouchi M, Tada H, Hirashima T, Asami K, Katakami N, Takada M (2010). Gefitinib versus cisplatin plus docetaxel in patients with non-small-cell lung cancer harbouring mutations of the epidermal growth factor receptor (WJTOG3405): an open label, randomised phase 3 trial. Lancet Oncol.

[6] Zhou C, Wu YL, Chen G, Feng J, Liu XQ, Wang C, Zhang S, Wang J, Zhou S, Ren S, Lu S, Zhang L, Hu C (2011). Erlotinib versus chemotherapy as first-line treatment for patients with advanced EGFR mutation-positive non-small-cell lung cancer (OPTIMAL, CTONG-0802): a multicentre, open-label, randomised, phase 3 study. Lancet Oncol.

[7] Rosell R, Carcereny E, Gervais R, Vergnenegre A, Massuti B, Felip E, Palmero R, Garcia-Gomez R, Pallares C, Sanchez JM, Porta R, Cobo M, Garrido P (2012). Erlotinib versus standard chemotherapy as first-line treatment for European patients with advanced EGFR mutation-positive non-small-cell lung cancer (EURTAC): a multicentre, open-label, randomised phase 3 trial. Lancet Oncol.

[8] Sequist LV, Yang JC, Yamamoto N, O’Byrne K, Hirsh V, Mok T, Geater SL, Orlov S, Tsai CM, Boyer M, Su WC, Bennouna J, Kato T (2013). Phase III study of afatinib or cisplatin plus pemetrexed in patients with metastatic lung adenocarcinoma with EGFR mutations. J Clin Oncol.

[9] Travis WD, Brambilla E, Nicholson AG, Yatabe Y, Austin JH, Beasley MB, Chirieac LR, Dacic S, Duhig E, Flieder DB, Geisinger K, Hirsch FR, Ishikawa Y (2015). The 2015 World Health Organization Classification of Lung Tumors: Impact of Genetic, Clinical and Radiologic Advances Since the 2004 Classification. J Thorac Oncol.

[10] Zehani A, Ayadi-Kaddour A, Marghli A, Maamouri H, Kassar L, Kilani T, El Mezni F (2014). [Sarcomatoid carcinoma of the lung: retrospective study of 28 cases]. Ann Pathol.

[11] Nash AD, Stout AP (1958). Giant cell carcinoma of the lung; report of 5 cases. Cancer.

[12] Hellstrom HR, Fisher ER (1963). Giant Cell Carcinoma of Lung. Cancer.

[13] Kumar M, Goel MM, Nupur (2015). Vertebral metastases from giant cell carcinoma of lung: Images in cytopathology. J Cytol.

[14] Fishback NF, Travis WD, Moran CA, Guinee DG, McCarthy WF, Koss MN (1994). Pleomorphic (spindle/giant cell) carcinoma of the lung. A clinicopathologic correlation of 78 cases. Cancer.

[15] Ginsberg SS, Buzaid AC, Stern H, Carter D (1992). Giant cell carcinoma of the lung. Cancer.

[16] Pelosi G, Gasparini P, Cavazza A, Rossi G, Graziano P, Barbareschi M, Perrone F, Barberis M, Takagi M, Kunimura T, Yamada T, Nakatani Y, Pastorino U (2012). Multiparametric molecular characterization of pulmonary sarcomatoid carcinoma reveals a nonrandom amplification of anaplastic lymphoma kinase (ALK) gene. Lung Cancer.

[17] Italiano A, Cortot AB, Ilie M, Martel-Planche G, Fabas T, Pop D, Mouroux J, Hofman V, Hofman P, Pedeutour F (2009). EGFR and KRAS status of primary sarcomatoid carcinomas of the lung: implications for anti-EGFR treatment of a rare lung malignancy. Int J Cancer.

[18] Beasley MB, Brambilla E, Travis WD (2005). The 2004 World Health Organization classification of lung tumors. Semin Roentgenol.

[19] Giroux Leprieur E, Antoine M, Vieira T, Duruisseaux M, Poulot V, Rabbe N, Belmont L, Gounant V, Lavole A, Milleron B, Lacave R, Cadranel J, Wislez M (2013). Clinical and molecular features in patients with advanced non-small-cell lung carcinoma refractory to first-line platinum-based chemotherapy. Lung Cancer.

[20] Vieira T, Girard N, Ung M, Monnet I, Cazes A, Bonnette P, Duruisseaux M, Mazieres J, Antoine M, Cadranel J, Wislez M (2013). Efficacy of first-line chemotherapy in patients with advanced lung sarcomatoid carcinoma. J Thorac Oncol.

[21] Tamura Y, Fujiwara Y, Yamamoto N, Nokihara H, Horinouchi H, Kanda S, Goto Y, Kubo E, Kitahara S, Tsuruoka K, Tsuta K, Ohe Y (2015). Retrospective analysis of the efficacy of chemotherapy and molecular targeted therapy for advanced pulmonary pleomorphic carcinoma. BMC Res Notes.

[22] Travis WD, Brambilla E, Noguchi M, Nicholson AG, Geisinger KR, Yatabe Y, Beer DG, Powell CA, Riely GJ, Van Schil PE, Garg K, Austin JH, Asamura H (2011). International association for the study of lung cancer/american thoracic society/european respiratory society international multidisciplinary classification of lung adenocarcinoma. J Thorac Oncol.

[23] Cai W, Lin D, Wu C, Li X, Zhao C, Zheng L, Chuai S, Fei K, Zhou C, Hirsch FR (2015). Intratumoral Heterogeneity of ALK-Rearranged and ALK/EGFR Coaltered Lung Adenocarcinoma. Journal of clinical oncology.

[24] Mitsudomi T, Yatabe Y (2007). Mutations of the epidermal growth factor receptor gene and related genes as determinants of epidermal growth factor receptor tyrosine kinase inhibitors sensitivity in lung cancer. Cancer Science.

[25] Rosell R, Moran T, Queralt C, Porta R, Cardenal F, Camps C, Majem M, Lopez-Vivanco G, Isla D, Provencio M, Insa A, Massuti B, Luis Gonzalez-Larriba J (2009). Screening for Epidermal Growth Factor Receptor Mutations in Lung Cancer. New England Journal Of Medicine.

[26] Arrieta O, Felipe Cardona A, Federico Bramuglia G, Gallo A, Campos-Parra AD, Serrano S, Castro M, Aviles A, Amorin E, Kirchuk R, Cuello M, Borbolla J, Riemersma O (2011). Genotyping Non-small Cell Lung Cancer (NSCLC) in Latin America. Journal Of Thoracic Oncology.

[27] Shi Y, Zhang L, Liu X, Zhou C, Zhang L, Zhang S, Wang D, Li Q, Qin S, Hu C, Zhang Y, Chen J, Cheng Y (2013). Icotinib versus gefitinib in previously treated advanced non-small-cell lung cancer (ICOGEN): a randomised, double-blind phase 3 non-inferiority trial. The Lancet Oncology.

[28] Lynch TJ, Bell DW, Sordella R, Gurubhagavatula S, Okimoto RA, Brannigan BW, Harris PL, Haserlat SM, Supko JG, Haluska FG, Louis DN, Christiani DC, Settleman J (2004). Activating mutations in the epidermal growth factor receptor underlying responsiveness of non-small-cell lung cancer to gefitinib. N Engl J Med.

[29] Paez JG, Janne PA, Lee JC, Tracy S, Greulich H, Gabriel S, Herman P, Kaye FJ, Lindeman N, Boggon TJ, Naoki K, Sasaki H, Fujii Y (2004). EGFR mutations in lung cancer: correlation with clinical response to gefitinib therapy. Science.

[30] Zou F, Xie G, Ma JA, Zhou DA, Jiang YI, Zheng JY (2015). Epidermal growth factor receptor mutation heterogeneity analysis of pulmonary sarcomatoid carcinoma successfully treated with erlotinib: A case report. Oncol Lett.

